# Necrotizing Soft Tissue Infection Secondary to Streptococcus constellatus, Actinomyces odontolyticus, and Gemella morbillorum in an Intravenous Drug User: A Case Report and Literature Review

**DOI:** 10.7759/cureus.37314

**Published:** 2023-04-08

**Authors:** Albertina Sebastian, Natasha Sebastian, Marutha Arulthasan, Ronald Simon, Jeffrey M Nicastro

**Affiliations:** 1 Surgery, Maimonides Medical Center, Brooklyn, USA; 2 Infectious Disease, Maimonides Medical Center, Brooklyn, USA

**Keywords:** actinomyces odontolyticus, iv drugs, gemella, necrotizing infections, soft tissue infections

## Abstract

A 53-year-old male with active IV heroin use presented with left upper extremity pain, erythema, swelling, and purulent foul-smelling drainage. Rapid diagnosis of necrotizing soft tissue infection (NSTI) was made based on clinical and radiologic findings. He was taken to the operating room for wound washouts and surgical debridements. The early microbiologic diagnosis was made based on intraoperative cultures. Successful treatment of NSTI in the setting of rare pathogens was achieved. The wound was ultimately treated with wound vac therapy, followed by primary delayed closure of the upper extremity and skin grafting of the forearm. We present a case of NSTI secondary to Streptococcus constellatus, Actinomyces odontolyticus, and Gemella morbillorum in an intravenous (IV) drug user, successfully treated with early surgical intervention.

## Introduction

Necrotizing soft tissue infections are commonly caused by gram-positive cocci. They can be defined according to their anatomical locations, such as Fournier gangrene) or the depth of infections, whether cellulitis, adipositis, fasciitis, or myositis [1]. Furthermore, necrotizing soft tissue infection (NSTI) can be classified into 4 types based on microbial etiology. Type I, the more common, is polymicrobial; Type II is mono microbial, most commonly associated with group A Streptococcus; Type III is specifically caused by Vibrio vulnificus; and Type IV is associated with fungal infections [2]. The Laboratory Risk Indicator for Necrotizing Fasciitis score, or more commonly LRINEC score, is a simple but robust score based on common laboratory values, such as total white cell count, hemoglobin, sodium, glucose, serum creatinine, and C-reactive protein (CRP). The LRINEC score allows practitioners to objectively measure the risk of necrotizing fasciitis in early clinical infections [3]. An immunocompromised state can lead to infection with various pathogens [4]. NSTIs are most commonly via bacterial colonization of skin wounds, including puncture wounds, such as in intravenous drug users. [5]. Our case demonstrates the variability in infectious pathogens associated with an immunocompromised state secondary to intravenous drug use.

We describe our experience with managing severe NSTI, resulting in the salvage of a critically infected limb. The patient agreed to have his case details and images published.

## Case presentation

**Figure 1:**
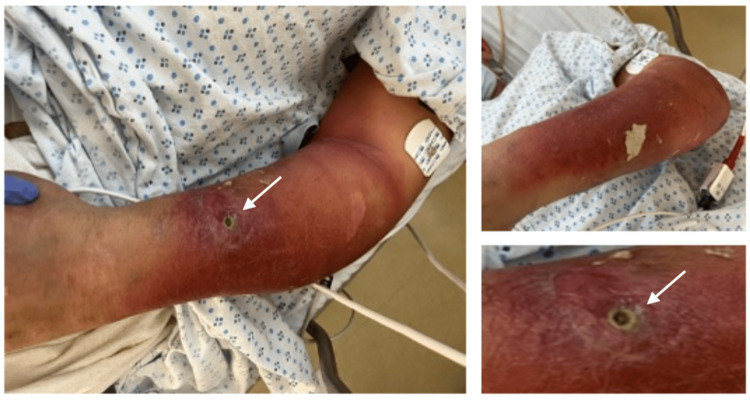
Left upper extremity upon initial presentation, with significant cellulitis and draining punctate wound (arrow).

A 53-year-old male presented to the emergency room to evaluate worsening left upper extremity (LUE) pain. The patient’s medical history was significant for an active Hepatitis C infection that was not being treated and a longstanding and active history of intravenous heroin use. During the interview, the patient admitted that symptoms began shortly after injecting himself into his left forearm, and the sterility of the needle was not disclosed. The pain quickly progressed over the next few days, including other concomitant symptoms, such as erythema, significant soft tissue swelling, and purulent drainage from an infected necrotic injection site (Figure 1).

At the initial evaluation, the patient was afebrile and normotensive. Laboratory values were significant for a lactic acid level of 3.0 and a sodium level of 130; however, the CRP was borderline, and the white blood cell count was within normal limits. The patient’s LRINEC Score was two, leaving him at low risk for necrotizing fasciitis. However, a slight elevation of the CRP would have increased his score to six, resulting in an intermediate risk for necrotizing fasciitis. Furthermore, clinical suspicion was high based on history, physical exam, and imaging findings (Table 1).

**Table 1:** LRINEC Score

	Value	Points
C-reactive protein (mg/L)	14.401	0
White blood cell count (x10,000/µL)	9.9	0
Hemoglobin (g/dL)	15.6	0
Sodium (mEq/L)	130	+2
Creatinine (µmol/L)	0.7	0

**Figure 2:**
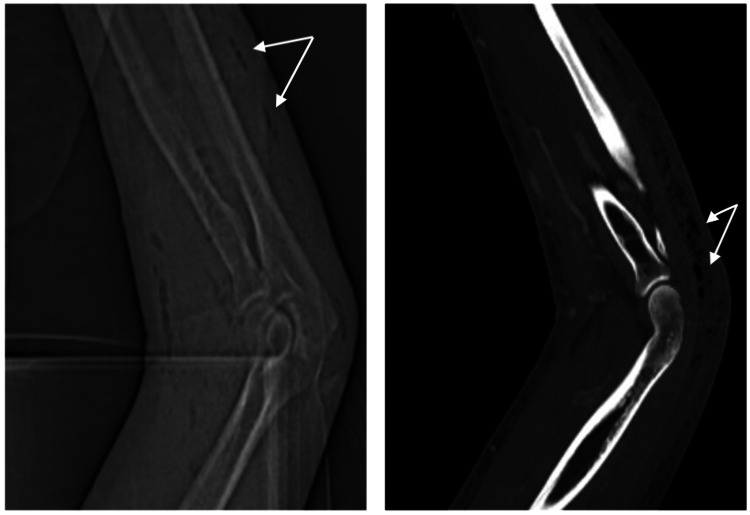
Radiographic imaging of the left upper extremity showing subcutaneous emphysema extending from the shoulder to the forearm (arrows) concerning necrotizing fasciitis.

The area of tenderness and erythema extended from the wrist to the upper arm and was warm to the touch. A potent, foul-smelling odor emanated from the punctate wound in the left forearm, draining tan-colored purulent fluid. Arterial supply and innervation were intact at the time of evaluation. Scout radiographic imaging of the left arm was significant for subcutaneous emphysema extending from the left shoulder to the forearm concerning NSTI (Figure 2).

**Figure 3:**
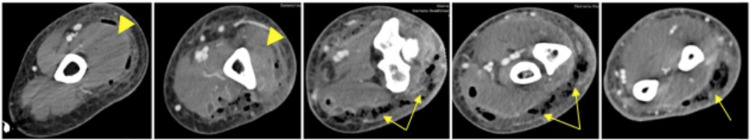
CT LUE with IV contrast, showing extensive subcutaneous emphysema/cellulitis with cutaneous emphysema with the gas-producing organism (arrows) and a fusiform subfascial abscess at the level of the posterior distal arm and extending proximally to the proximal arm (arrowheads). LUE: left upper extremity

Subsequent cross-sectional imaging of the upper extremity with IV contrast confirmed extensive subcutaneous emphysema/cellulitis and cutaneous emphysema, presumably from a gas-producing organism, as well as a fusiform subfascial abscess at the level of the posterior distal arm and extending proximally to the proximal arm (Figure 3).

Given the overall clinical evidence of rapidly worsening NSTI, a decision was made to take the patient to the operating room for wide incisional drainage and debridement.

The incision was initially performed at the forearm level, revealing pockets of foul-smelling purulent fluid extending from the wrist to the midarm. The incision was then carried out proximally towards the posterior upper arm. There was evidence of skin and subcutaneous fat necrosis; however, the fascia and muscle were noted to be intact and relatively healthy. A member of the Orthopedic Surgery team was present intraoperatively, and no involvement of the ulnohumeral joint was noted. Tissue cultures and a swab culture were sent for analysis. The wound was copiously irrigated, and a wet-to-dry dressing using a gauze roll was applied. Infectious Disease evaluated the patient, and he was placed on IV Piperacillin/Tazobactam (4500mg every 8 hours) and IV Linezolid (600mg every 12 hours) for broad-spectrum coverage postoperatively.

**Figure 4:**
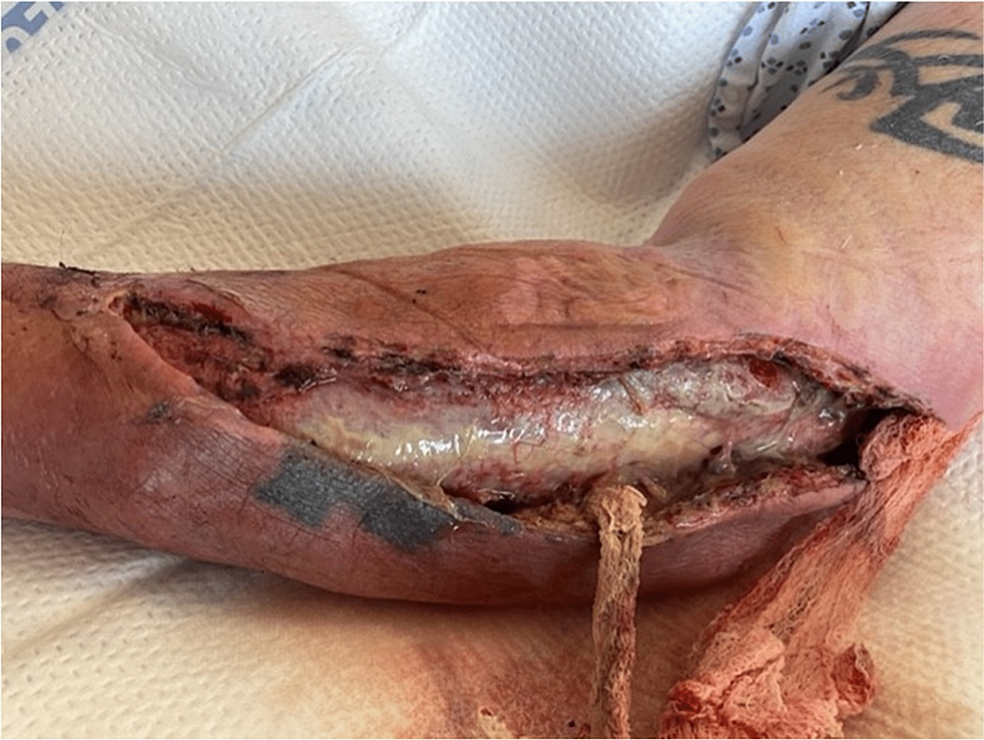
Surgical wound on POD1 from index procedure, demonstrating significant purulent drainage with concern for muscle involvement.

Overnight, the patient remained afebrile and normotensive. His heart rate remained at approximately 100 bpm. On postoperative day 1, laboratory values were significant for a CO_2_ of 14 and a WBC of 8.3 with a left shift. The patient complained of significant pain during an exam. The dressing was taken down, and upon removal of the Kerlix packing, there was much foul-smelling, purulent fluid. In addition, the skin of the left forearm appeared to be necrotic, and there was a concern for invasion of infection into the musculature (Figure 4).

**Figure 5:**
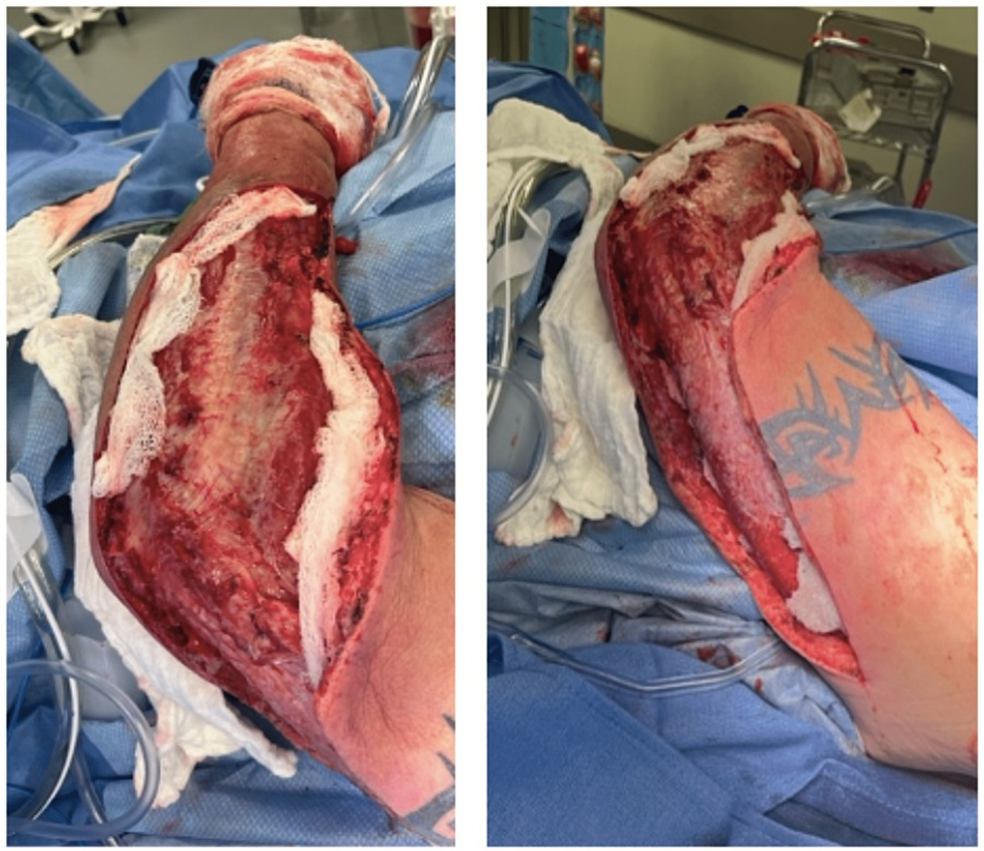
Surgical wound during a second intervention, post debridement, and washout.

A decision was made to take the patient back to the operating room promptly. Intraoperatively, the forearm incision was extended approximately three centimeters toward the wrist. Both medial and lateral forearm flaps were then debrided, with excision of approximately 40 cm^2^ and 60 cm^2^ of skin and soft tissue, respectively. The skin edges appeared well-perfused and were actively bleeding. Two areas of patchy necrosis were noted on the fascia overlying the muscle, which was sharply debrided and sent for permanent pathology and culture. The muscle itself was viable. The superior pole of the upper arm incision was then extended by approximately four centimeters towards the shoulder, and blunt dissection of the soft tissue of the underlying fascia allowed for drainage of additional purulent fluid. All skin and subcutaneous tissue in this area appeared viable. A four-centimeter counter incision was then made along the sagittal axis of the medial forearm. The entirety of the wound was washed out copiously and irrigated with five liters of normal saline using a pulse irrigator. The wound was packed with a wet-to-dry dressing using a gauze roll (Figure 5).

**Figure 6:**
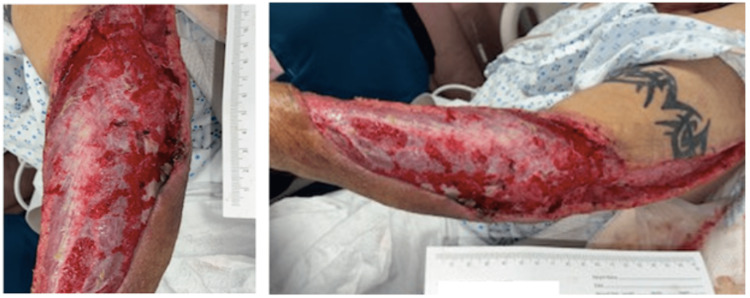
Surgical wound on POD5 from the index procedure and POD4 from the takeback.

Cultures from the index procedure grew moderate Streptococcus constellatus and moderate Actinomyces odontolyticus. Cultures from the takeback surgery also grew moderate Streptococcus constellatus; however, evidence of moderate Gemella morbillorum was also found within this culture specimen. After reevaluation by Infectious Disease, the patient’s antibiotic regimen was converted to IV Ampicillin/Sulbactam based on susceptibilities. Daily wet-to-dry dressings were carried out, and appropriate healing was noted. On postoperative day four from the takeback surgery, there was evidence of adequate blood supply within the wound bed and tissue granulation (Figure 6).

**Figure 7:**
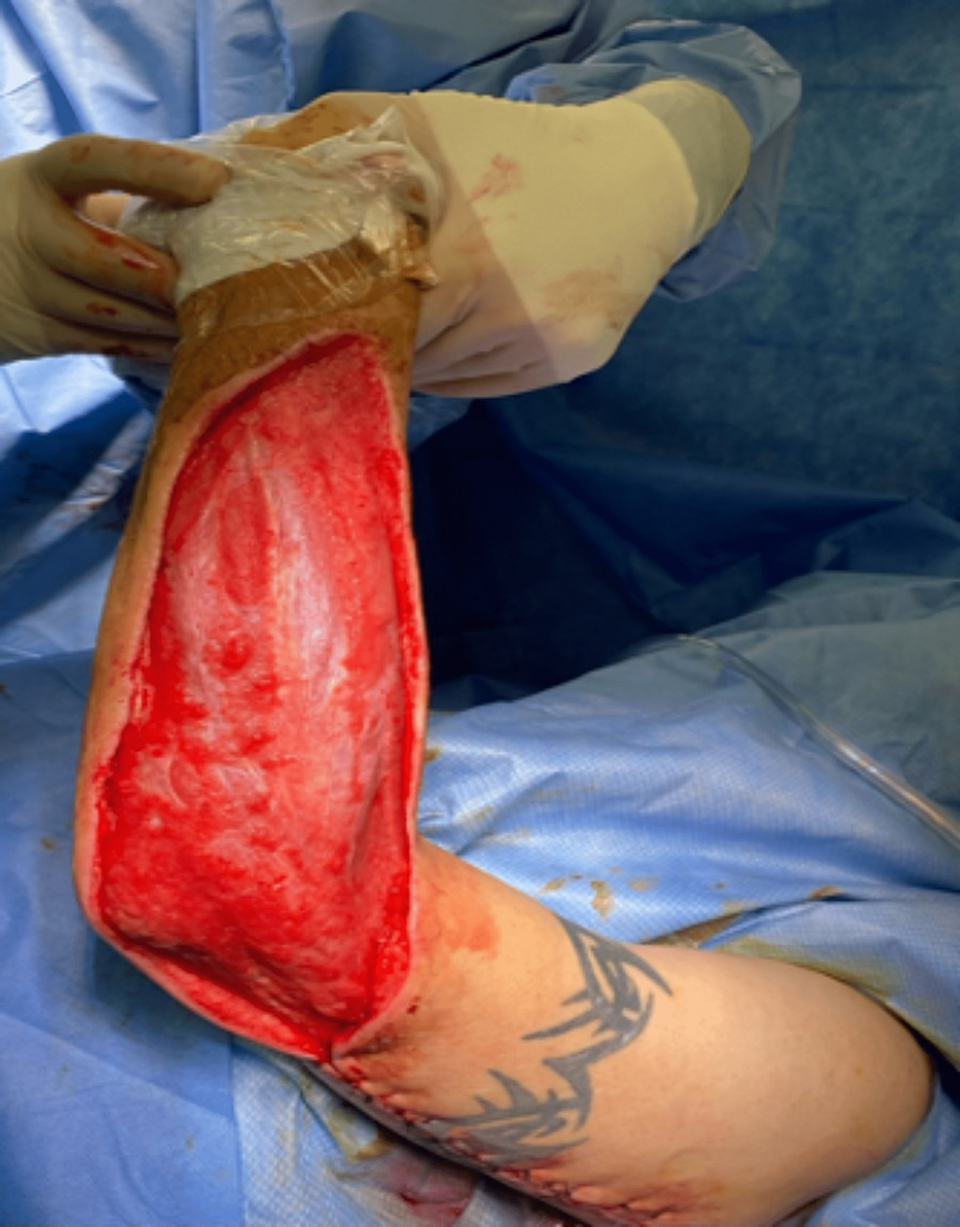
Surgical wound after reapproximating upper extremity skin flaps.

The decision was made to convert to wound vac therapy. Approximately two weeks postoperatively, the patient was taken back to the operating room for the first stage of wound closure. The upper arm skin was reapproximated with an interrupted parallel mattress and simple interrupted sutures using 2-0 and 3-0 Nylon sutures. The forearm base was noted to be too wide for delayed primary closure due to the previous extent of skin excision. The wound borders spanned approximately 25 cm in length, 8cm in width, and 1 cm in depth (Figure 7).

**Figure 8:**
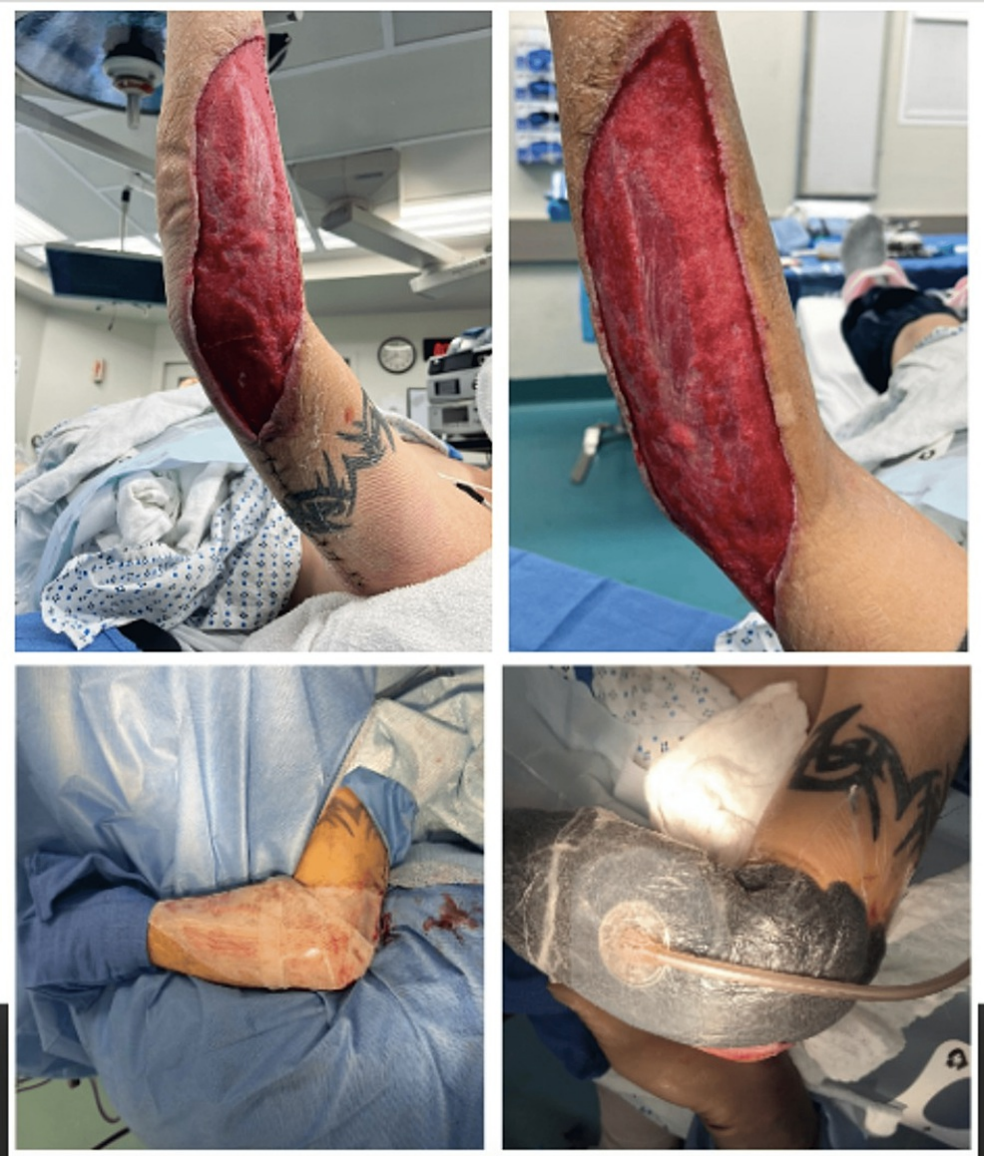
Wide forearm wound base (above) pre-skin grafting and (below) post-skin grafting with overlying vac therapy.

A wound vac was replaced over the forearm. Plastic Surgery was consulted regarding wound closure, and skin grafting was recommended. Roughly one week after his most recent takeback surgery, the patient was returned to the operating room for final closure. Epidermis and dermis were harvested from the patient’s right thigh, and a split-thickness skin graft was applied to the right forearm and fixed with surgical skin staples (Figure 8).

**Figure 9:**
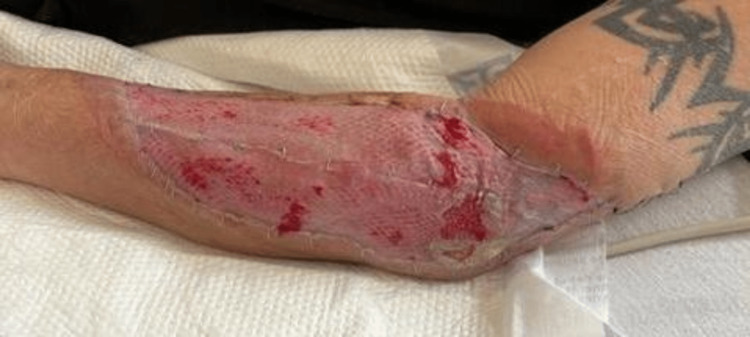
Forearm wound 6 days post split-thickness skin grafting.

Infectious disease followed the patient throughout his hospital course, and he remained on IV ampicillin/sumbactam perioperatively. Per recommendations, the patient will remain on oral amoxicillin/clavulanic acid 875mg twice daily for six to 12 months post-discharge to adequately treat his soft tissue infection. Given his active heroin use upon initial presentation, the patient was discharged to a rehabilitation facility after adequately healing his infected wound (Figure 9).

Follow-up with General Surgery and Infectious Disease was arranged to ensure adequate resolution of the affected left upper extremity; however, the patient was lost to follow-up.

## Discussion

Skin and soft tissue infections (SSTI) most commonly occur when there is a breach in the protective skin barrier. This allows for normal flora and other infectious pathogens to penetrate the skin. They can involve the epidermis, dermis, or subcutaneous tissue. SSTIs can be classified as simple or complicated, and the latter can be subclassified as necrotizing or non-necrotizing [4]. Unlike simple infections that tend to be confined to the skin and superficial soft tissues, complicated infections involve deeper structures, including deep abscesses, decubitus ulcers, necrotizing fasciitis, and Fournier’s gangrene. The most frequently affected areas for SSTIs include the arms, legs, and buttocks. Less commonly affected areas include the neck, groin, and torso. Simple SSTIs typically present signs of local inflammation, such as erythema, edema, warmth, pain, and induration. Patients with necrotizing soft tissue infections (NSTI) are found to have pain out of proportion to the physical exam, rapid progression of the infection, hemorrhagic or bullous changes, and crepitus, which indicates gas in the subcutaneous tissue.

As previously described, NSTIs can be classified into 4 types based on their microbial etiology. Type I infections are polymicrobial, caused by gram-positive and gram-negative aerobic and anaerobic bacteria. Type II is predominantly caused by Streptococcus pyogenes (GAS) or Staphylococcus aureus, which includes MRSA. Management of these infections is based on their severity and is determined by the acuity of the infection, the absence or presence of purulence, and the type of infection. Vancomycin, metronidazole, and third-generation or fourth-generation cephalosporin allow for broad-spectrum coverage of NSTIs. If Streptococcus pyogenes is the causative agent of an NSTI, Clindamycin may be added for coverage of its antitoxin [4,6]. Type III is caused by Vibro Vulnificus, and Type IV by fungal etiology in immunocompromised patients [2].

A vital population to consider when studying cases of NSTIs is people who inject drugs (PWID). Significant risks are associated with IV drug administration, including septicemia, infective endocarditis, and NSTIs, which may affect the bones and joints [7]. In addition to this population's generalized poor socioeconomic status, the drug preparation and administration process poses a considerable risk factor for infection. Infection can occur by direct bacterial inoculation at the injection site or bacterial contamination of the drug or the injection equipment. Behavioral practices of PWID also increase the risk of infection transmissions, such as needle licking, shared injection equipment, and frequent injections [6]. This population also includes a subset of immunocompromised patients, most commonly with human immunodeficiency virus (HIV) infection, diabetes mellitus (DM), or Hepatitis C. A study by Dahlman et al. showed that the use of heroin had a higher risk of association with SSTIs compared to other drugs, such as amphetamines. More specifically, black tar heroin was associated with greater vein loss and soft tissue abscesses than heroin in its powdered form [8].

After an extensive literature review, our patient may seem like a typical case of NSTI in the setting of IV drug use. However, the pathogens which grew from his cultures make this case unique. Initial cultures grew Streptococcus constellatus and Actinomyces odontolyticus. Streptococcus constellatus is a gram-positive facultative anaerobic organism of the Streptococcus milleri family. The virulence of this organism is known to increase in the setting of coinfection with another bacteria significantly [9]. This pathogen is rare; however, a few cases have been reported in the literature [10]. Actinomyces odontolyticus are filamentous, anaerobic gram-positive bacilli. They are common pathogens of the normal flora of the oropharynx, the gastrointestinal tract, and the urogenital tract.

Abscesses secondary to this pathogen can translocate to adjacent sites via hematogenous seeding, most commonly to the central nervous system through the cervicofacial structures, such as fascial planes and sinus tracts [11]. Although cases of purulent infections by Actinomyces odontolyticus have been reported [12,13], their involvement in NSTIs via inoculation by IV drug use is rare. When questioning our patient about his behavioral habits while injecting, he denied licking the needle before injection, which decreases or eliminates an oropharyngeal contamination source. However, this may be subjective to recall bias. His second set of wound cultures grew Gemella morbillorum, a facultative anaerobe gram-positive coccus. It is part of the normal flora of the respiratory, urinary, and gastrointestinal tracts in humans. The literature reports a few infections caused by this organism, including endocarditis, neurologic infections, and NSTI secondary to a pustule [14]. Mechanisms of infections can include endoscopic procedures; however, reports of infection in PWID remain sparse (table 2).

**Table 2:** Review of publications cited

Name of paper	Authors, Year of Publication	Microorganisms Involved	Treatment	Outcome
Necrotizing myositis of the deltoid following intramuscular injection of anabolic steroid	Grant, 2010 [9]	Gemella morbillorum and Veillonella (pus) Dialister pneumosintes (Blood)	Benzylpenicillin and Clindamycin	Alive, preserved shoulder function
Tricuspid valve endocarditis due to a moderately susceptible Streptococcus constellatus: Persistent bacteremia and fatal outcome despite penicillin Plus gentamicin therapy	Baran Jr., 2009 [10]	Streptococcus constellatus	Penicillin and Gentamicin	Deceased
Actinomyces odontolyticus causing meningitis and cervical abscess	Jain, 2021 [12]	Actinomyces odontolyticus and Streptococcus intermedius	Vancomycin, Cefepime, and Metronidazole Followed by Ceftriaxone 2g IV Q12H x 6 wks	Alive
		Readmission: Actinomyces odontolyticus	Readmission: Ceftriaxone 2g IV Q12H and Metronidazole 500mg IV Q8H x 4 wks Followed by: Amoxicillin-Clavulanic acid 875mg PO BID x 1 yr	
Actinomyces odontolyticus: Rare Etiology for Purulent Pericarditis	Mack, 2021 [13]	Actinomyces odontolyticus	Piperacillin-tazobactam, Vancomycin, and Ciprofloxacin	Deceased
Gemella morbillorum is a source bacteria for necrotizing fasciitis of the torso	Romero-Velez, 2020 [14]	Gemella morbillorum	Piperacillin-tazobactam, Vancomycin, and Clindamycin Followed by: Clindamycin PO	Alive
g, Gram; IV, Intravenous; Q12H, every 12 hours; x, for; wks, weeks; PO, by mouth.				

Our patient demonstrated a type one NSTI. Despite remaining afebrile with nearly normal blood work, rapid diagnosis based on clinical and radiologic findings was imperative to his successful treatment. A case series by Takahashi et al. showed that fevers are not always present in the setting of NSTIs. They found that less than 10% of patients had documented fevers upon presentation, and only approximately one-third reported having subjective fevers [15].

## Conclusions

Despite NSTIs occurring commonly in PWID, our case demonstrates the variability in infectious pathogens associated with an immunocompromised state. Although acuity, virulence factor, and disease progression differs amongst pathogens, we conclude that early microbiologic diagnosis and expeditious surgical intervention, and source control remain the gold standard for treating necrotizing soft tissue infections.
